# Release of nickel and chromium ions from orthodontic wires following the use of teeth whitening mouthwashes

**DOI:** 10.1186/s40510-018-0203-7

**Published:** 2018-02-05

**Authors:** AmirHossein Mirhashemi, Sahar Jahangiri, MohammadJavad Kharrazifard

**Affiliations:** 1 Orthodontic Department, School of Dentistry, Tehran University of Medical Sciences, International Campus, Laser Research Center of Dentistry, Tehran, Iran; 2 School of Dentistry, Tehran University of Medical Sciences, Tehran, Iran; 3 Dental Research Center, Dentistry Research Institute, Tehran University of Medical Sciences, Tehran, Iran

**Keywords:** Ions, Nickel, Chromium, Mouthwash, Wire

## Abstract

**Background:**

Corrosion resistance is an important requirement for orthodontic appliances. Nickel and chromium may be released from orthodontic wires and can cause allergic reactions and cytotoxicity when patients use various mouthwashes to whiten their teeth. Our study aimed to assess the release of nickel and chromium ions from nickel titanium (NiTi) and stainless steel (SS) orthodontic wires following the use of four common mouthwashes available on the market.

**Methods:**

This in vitro, experimental study was conducted on 120 orthodontic appliances for one maxillary quadrant including five brackets, one band and half of the required length of SS, and NiTi wires. The samples were immersed in Oral B, Oral B 3D White Luxe, Listerine, and Listerine Advance White for 1, 6, 24, and 168 h. The samples immersed in distilled water served as the control group. Atomic absorption spectroscopy served to quantify the amount of released ions.

**Results:**

Nickel ions were released from both wires at all time-points; the highest amount was in Listerine and the lowest in Oral B mouthwashes. The remaining two solutions were in-between this range. The process of release of chromium from the SS wire was the same as that of nickel. However, the release trend in NiTi wires was not uniform.

**Conclusions:**

Listerine caused the highest release of ions. Listerine Advance White, Oral B 3D White Luxe, and distilled water were the same in terms of ion release. Oral B showed the lowest amount of ion release.

## Background

Fixed orthodontic wires and brackets are made of stainless steel (SS) and nickel titanium (NiTi) which consists of chromium, cobalt, nickel, and titanium [1, 2]. All metal components undergo corrosion in the oral environment due to chemical, mechanical, thermal, microbiological, and enzymatic changes leading to release of ions [3]. Electrochemical processes play an important role in corrosion when two alloys and one medium as the electrolyte are present. The alloy with lower resistance against corrosion acts as the anode and dissolves into the electrolyte, and ions are then released.

Release of ions can cause discoloration of the adjacent soft tissues, allergic reactions, or pain. The released ions can also cause toxic and biological side effects [4, 5]. Nickel can cause allergic reactions [2]. Allergic reactions to chromium released from orthodontic components have also been reported [6, 7]. Aside from the allergic reactions, release of ions may even cause cytotoxic effects, mutagenesis, and carcinogenesis [8, 9].

Tooth color and appearance of the teeth significantly affect the self-esteem and social self-image [10]. Thus, many patients seek tooth bleaching which can be performed in the office or at home [11, 12]. Several over-the-counter bleaching products are available on the market. These products contain lower percentage of bleaching agents compared to in-office bleaching agents. These products, available in the form of mouthwashes, do not require a prescription and are usually used daily over a period of 2 weeks [12].

Several studies have assessed the release of ions from orthodontic wires and brackets in contact with the saliva and fluoridated mouthwashes. A previous study stated that orthodontists must be careful when prescribing mouthwashes for orthodontic patients with orthodontic appliances [13]. Release of ions increases with increase in the pH [14]. It has been reported that the amount of ions released from NiTi wires is higher than that released from SS wires [15].

Our study aimed to assess the release of nickel and chromium ions during immersion in Listerine, Oral B, Oral B 3D White Luxe, and Listerine Advance White.

## Methods

In this in vitro, experimental study, release of nickel and chromium ions from a complex of one 0.016-in wire (NiTi or SS), five brackets, and one band (American Orthodontics, Sheboygan USA) immersed in different mouthwashes was evaluated. Four different mouthwashes (Listerine, Oral B, Oral B 3D White Luxe, and Listerine Advanced White) and distilled water as the control group were used in this study (Table 1). A total of 120 wires were obtained (60 NiTi and 60 SS wires). The wires were cleaned using acetone, rinsed with distilled water, and dried. Orthodontic components for one maxillary quadrant including five brackets, one band and wire were placed in a screw-top vial (10 mm volume and a total of 120 samples). Since sample size was 3 in each group [16] and the assessments were made at four time-points, a total of 60 wires were used. The vials were divided into five groups each with 24 samples. In each group, 10 mL of the respective solutions were poured (12 containers containing SS and 12 containing NiTi wires).

**Table 1:** Details of the tested whitening mouthrinses

Mouthwash	Ingredients	Manufacturer
Oral B	Aqua, glycerin, polysorbare 20, aroma, methyl paraben, cetylpyridinium chloride, sodium fluoride, sodium saccharin, sodium benzoate, propylparaben, Cl 42051, Cl 47005	Procter & Gamble, Weighbridge, UK
Oral B 3D White Luxe	Aqua, glycerin, alcohol, aroma, methyl paraben, poloxamer 407, cetylpyridinium chloride, sodium fluoride, sodium saccharin, propyl paraben, Cl 42051, Cl 47005	Procter & Gamble, Weighbridge, UK
Listerine	Aqua, sorbitol, propylene glycol, aroma, poloxamer 407, sodium sulfate, phosphoric acid, eucalyptol, methyl salicylate, thymol, sodium benzoate, sodium saccharin, menthol, sucralose, disodium phosphate	Johnson & Johnson S.P.A, Pomezia, Italy
Listerine Advanced White	Aqua, alcohol, sorbitol, tetrapotassium pyrophosphate, pentasodium triphosphate, citric acid, poloxamer 407, sodium benzoate, eucalyptol, thymol, sodium saccharin, sodium fluoride, menthol, sucralose, tetrasodium pyrophosphate, propylene glycol, sucralose, aroma, disodium phosphate	Johnson & Johnson S.P.A, Pomezia, Italy

The samples were incubated at 37 °C for 1, 6, and 12 h and 1 week. At each time-point, 15 vials of each type of wire were randomly chosen and removed from the incubator. The wires were removed from the solution, and then, one drop of 65% nitric acid was added to stabilize the released ions. Calibration was done by measuring the amount of nickel released using atomic absorption spectroscopy. Univariate ANOVA served to assess the effect of mouthwashes on release of nickel and chromium ions from the orthodontic wires. Since the interaction of the variables was significant, one-way ANOVA and Tukey’s HSD test were used to assess the effect of each variable.

## Results

The results showed an increase in release of nickel and chromium ions in all mouthwashes and at all time-points from both types of wires (Tables 2, 3, 4, 5, 6, and 7). The release of nickel ions from both types of wires at all time-points was the highest in Listerine and the lowest in Oral B. The other two solutions were somewhere between this range. Also, the release of chromium from SS wires was similar to that of nickel from NiTi with the difference that chromium release did not follow a uniform trend as in NiTi wires.

**Table 2:** Release of chromium ions from NiTi wires at various time-points

	L&D	O&D	LB&D	OB&D	L&O	L&LB	L&OB	O&LB	O&OB	OB&LB
1 h	0.001	0.00	0.00	0.75	0.42	0.42	0.005	1.00	0.08	0.08
6 h	0.00	0.24	0.48	0.48	0.004	0.002	0.002	0.97	0.97	1.00
24 h	0.001	0.13	0.001	0.01	0.02	1.00	0.28	0.02	0.53	0.28
168 h	0.00	0.00	0.00	0.00	0.02	0.00	0.00	0.00	0.00	0.00

*D* distilled water, *L* Listerine, *O* Oral B, *LB* Listerine Advance White, *OB* Oral B 3D White Luxe

**Table 3:** Release of chromium ions from SS wires at various time points

	L&D	O&D	LB&D	OB&D	L&O	L&LB	L&OB	O&LB	O&OB	OB&LB
1 h	0.16	0.85	0.16	0.85	0.03	1.00	0.03	0.03	1.00	0.03
6 h	0.02	0.70	0.05	0.96	0.003	0.96	0.05	0.008	0.35	0.14
24 h	0.02	0.06	0.13	0.9	0.00	0.82	0.06	0.001	0.02	0.28
168 h	0.003	0.003	0.01	0.001	0.00	0.82	0.82	0.00	0.00	0.28

*D* distilled water, *L* Listerine, *O* Oral B, *LB* Listerine Advance White, *OB* Oral B 3D White Luxe

**Table 4:** Release of nickel ions from NiTi wires at various time-points

	L&D	O&D	LB&D	OB&D	L&O	L&LB	L&OB	O&LB	O&OB	OB&LB
1 h	0.00	0.29	0.002	0.29	0.00	0.00	0.00	0.03	1.00	0.03
6 h	0.00	0.53	0.00	0.04	0.00	0.00	0.00	0.00	0.44	0.00
24 h	0.00	0.00	0.00	0.00	0.00	0.00	0.00	0.00	0.13	0.00
168 h	0.00	0.99	0.92	0.89	0.00	0.00	0.00	0.98	0.98	1.00

*D* distilled water, *L* Listerine, *O* Oral B, *LB* Listerine Advance White, *OB* Oral B 3D White Luxe

**Table 5:** Release of nickel ions from SS wires at various time-points

	L&D	O&D	LB&D	OB&D	L&O	L&LB	L&OB	O&LB	O&OB	OB&LB
1 h	0.00	0.88	0.00	0.002	0.00	0.00	0.00	0.00	0.007	0.001
6 h	0.00	0.7	0.00	0.02	0.00	0.00	0.00	0.00	0.00	0.008
24 h	0.00	1.00	0.00	0.00	0.00	0.00	1.00	0.00	0.00	0.00
168 h	0.00	0.03	0.42	0.00	0.00	0.00	0.97	0.42	0.00	0.00

*D* distilled water, *L* Listerine, *O* Oral B, *LB* Listerine Advance White, *OB* Oral B 3D White Luxe

**Table 6:** Release of nickel ions from NiTi and SS wires at various time-points

Ni (mean + SD)					
		1 h	6 h	24 h	168 h
Distilled water	NITI	10 ± 0	16 ± 2.8	20 ± 0	23.3 ± 2.8
	SS	10 ± 0	13.3 ± 2.8	21.6 ± 2.8	53.3 ± 2.8
Listerine	NITI	276 ± 20.8	576 ± 25.1	3180 ± 20	11,500 ± 500
	SS	53.3 ± 2.8	61.6 ± 2.8	63.3 ± 2.8	83.3 ± 2.8
Oral B	NITI	26.6 ± 2.8	31.6 ± 2.8	81.6 ± 2.8	85 ± 5
	SS	11.6 ± 2.8	15 ± 0	21.6 ± 2.8	43.3 ± 2.8
Listerine Advance White	NITI	55 ± 5	148.3 ± 2.8	168.3 ± 2.8	170 ± 5
	SS	31.6 ± 2.8	35 ± 5	43.3 ± 2.8	48.3 ± 2.8
Oral B 3D White Luxe	NITI	26.6 ± 2.8	48.3 ± 2.8	101.6 ± 2.8	185 ± 5
	SS	20 ± 0	23.3 ± 2.8	63.6 ± 3.2	85 ± 5

*SD* standard deviation

**Table 7:** Release of chromium ions from NiTi and SS wires at various time-points

Cr (mean + SD)					
		1 h	6 h	24 h	168 h
Distilled water	NITI	16.6 ± 5.7	26.6 ± 5.7	31.6 ± 2.8	33.3 ± 2.8
	SS	20 ± 0	23.3 ± 2.8	33.3 ± 2.8	45 ± 5
Listerine	NITI	33.3 ± 2.8	48.3 ± 2.8	51.6 ± 2.8	61.6 ± 2.8
	SS	28.3 ± 2.8	33.3 ± 2.8	45 ± 5	61.6 ± 2.8
Oral B	NITI	28.3 ± 2.8	33.3 ± 2.8	40 ± 5	75 ± 5
	SS	16.6 ± 5.7	20 ± 0	23.3 ± 2.8	28.3 ± 2.8
Listerine Advance White	NITI	28.3 ± 2.8	31.6 ± 2.8	51.6 ± 2.8	140 ± 5
	SS	28.3 ± 2.8	31.6 ± 2.8	41.6 ± 2.8	58.3 ± 2.8
Oral B 3D White Luxe	NITI	20 ± 0	31.6 ± 2.8	45 ± 5	175 ± 5
	SS	16.6 ± 5.7	25 ± 5	35 ± 5	65 ± 5

*SD* standard deviation

## Discussion

Our results showed the release of nickel and chromium ions from orthodontic wires in mouthwashes but not in distilled water. This release from SS wires was less than that from NiTi wires. At all time-points, release of both ions in Listerine was the highest from both wires. However, the rate of release of nickel from SS wire at 24 h and 1 week in Listerine was similar to that in Oral B 3D White Luxe. The amount of released chromium ions at 1 week from SS wire in Oral B 3D White Luxe was higher than that in Listerine. It seems that Oral B 3D White Luxe caused greater release of ions at 1 week. Also, our results showed that the lowest release of ions occurred in Oral B, although the release of chromium ions from NiTi wire did not completely follow this trend.

Since it has been reported that nickel and chromium ions may have toxic or carcinogenic effects [17, 18], many orthodontists became concerned. Barret et al. in 1993 assessed the release of chromium and nickel ions from NiTi and SS wires at 1, 7, 14, 21, and 28 days and reported that release of nickel ions after 1 week and chromium after 2 weeks reached the maximum level [3]. Thus, 1 week was considered as the longest time of immersion in our study. Also, the release of nickel ions from both wires was 37 times greater than that of chromium [3]. Gursoy et al. in 2005 compared the release of ions from NiTi wires during 45 days, and the results indicated significant increase in concentration of these ions especially nickel and chromium released from the wires [19]. Thus, new wires were used for various immersion periods in our study.

Matos de Souza et al. in 2008 assessed the release of nickel, chromium, and iron ions from three types of brackets in the saliva in vivo and showed an abrupt increase in concentration of nickel and chromium after immersion of orthodontic components in all three groups. They found no significant difference in release of these ions among the three types of brackets [20]. In our study, this value reached maximum at 1 week, and a significant difference was found between the two types of wires in this respect.

Kuhta et al. in 2009 assessed the release of ions from three types of wires namely Thermo NiTi, SS, and NiTi immersed in artificial saliva with a pH of 3.5 and 6.75 for 1, 7, 14, and 28 days. In their study, the highest release of nickel from SS wires occurred at a pH of 6.75, but at a pH of 3.5, the release rate was almost the same in the three types of wires and was significantly higher than that at a pH of 6.75 [21]. It seems that the difference in the pH of Listerine (4 ± 0.5) and other solutions (7 ± 0.5) was responsible for the difference in release of ions. They also found that in the first 7 days of immersion, ion release occurred more intensely compared to other time-points; therefore, we studied 1 week duration of immersion. Momeni Danai et al. in 2011 assessed the release of nickel and chromium ions from SS brackets in chlorhexidine, Persica, and Oral B; they noticed that this rate, being close to the threshold, was higher in chlorhexidine compared to that in other mouthwashes. Moreover, this rate was higher in distilled water [22]. In our study, nickel release was noticeable from SS wires in Oral B at 24 h and 1 week, and in Listerine and Advance White at 1 week. Chromium release was noted in Oral B at all time-points and in Oral B 3D White Luxe at 1 h. Mikulewicz et al. in 2012 assessed the release of ions from SS wires in artificial saliva and found that this value reached the maximum safe threshold for nickel ions at 30 days, but the release of chromium was lower than this rate [23]. In our study, the amount of nickel released in Listerine at all time-points was close to the threshold, but the release of chromium was not concerning.

Senkutvan et al. in 2014 assessed the release of nickel ions from all four types of SS, NiTi, CuNiTi, and implanted NiTi wires in artificial saliva and found that ion release in the first 7 days was more severe compared to that in other time-points. Also, they found that this value was the highest for NiTi and the lowest for SS wires [24]. Our results confirmed their findings.

Jamilian et al. in 2014 evaluated ion release from NiTi and SS wires in Oral B and Orthokin mouthwashes and noticed that this value was lower than the safe threshold. Also, NiTi wires released more ions than did SS wires [15]. This finding was also confirmed in our study.

Tahmasebi et al. in 2015 evaluated the release of ions from wires and brackets in Oral B fluoride mouth rinse and found that NiTi wires had the highest release of nickel ions and SS wires had the highest release of chromium [25]. In our study, the release of both ions from NiTi wires was higher than that from SS wires, although some differences were observed at different time-points. The results of our study showed that the release of ions from both wires in Oral B 3D White Luxe, Listerine Advance White, and Oral B was within the safe threshold [14, 26, 27], but the release of nickel from NiTi wires in Listerine was higher than this threshold probably due to the low pH of this solution. Thus, it seems that more attention must be paid when prescribing this rinse for patients with NiTi wires. Due to differences in physiological conditions of the oral cavity [28, 29] and in vitro conditions, in vivo studies on this subject and use of artificial saliva are recommended to obtain more accurate results. Also, due to the variability in rinses available over the counter, future studies using other mouthwashes are recommended.

## Conclusions

It seems that Listerine causes the highest release of ions, and Listerine Advance White, Oral B 3D White Luxe, and distilled water were similar in terms of ion release. Oral B caused the lowest release of ions.
